# Hemozoin “knobs” in *Opisthorchis felineus* infected liver

**DOI:** 10.1186/s13071-015-1061-5

**Published:** 2015-09-17

**Authors:** Alexandra G. Pershina, Irina V. Saltykova, Vladimir V. Ivanov, Ekaterina A. Perina, Alexander M. Demin, Oleg B. Shevelev, Irina I. Buzueva, Anton K. Gutakovskii, Sergey V. Vtorushin, Ilya N. Ganebnykh, Victor P. Krasnov, Alexey E. Sazonov, Ludmila M. Ogorodova

**Affiliations:** Siberian State Medical University, 2, Moskovsky trakt, 634050 Tomsk, Russia; Postovsky Institute of Organic Synthesis, UB RAS, 22, S. Kovalevskoy St, 620137 Yekaterinburg, Russia; Institute of Cytology and Genetics, SB RAS, 10, Lavrentyev Ave, 630090 Novosibirsk, Russia; Federal State Budgetary Scientific Institution “Scientific Research Institute of Physiology and Basic Medicine”, 4, Timakova St, 630117 Novosibirsk, Russia; Rzhanov Institute of Semiconductor Physics, SB RAS, 13, Lavrentyev Ave, Novosibirsk, 630090 Russia; National Research Tomsk Polytechnic University, 30, Lenina Ave, Tomsk, 634050 Russia; National Research Tomsk State University, 36, Lenina Ave, Tomsk, 634050 Russia

**Keywords:** Hemozoin, Trematode, *Opisthorchis felineus*, Bile duct ectasia, Magnetic resonance imaging (MRI), Transmission electron microscopy (TEM)

## Abstract

**Background:**

Hemozoin is the pigment produced by some blood-feeding parasites. It demonstrates high diagnostic and therapeutic potential. In this work the formation of co-called hemozoin “knobs” – the bile duct ectasia filled up by hemozoin pigment - in *Opisthorhis felineus* infected hamster liver has been observed.

**Methods:**

The *O. felineus* infected liver was examined by histological analysis and magnetic resonance imaging (MRI). The pigment hemozoin was identified by Fourier transform infrared spectroscopy and high resolution electrospray ionization mass spectrometry analysis. Hemozoin crystals were characterised by high resolution transmission electron microscopy.

**Results:**

Hemozoin crystals produced by O. *felineus* have average length 403 nm and the length-to-width ratio equals 2.0. The regurgitation of hemozoin from parasitic fluke during infection leads to formation of bile duct ectasia. The active release of hemozoin from O. *felineus* during *in vitro* incubation has also been evidenced. It has been shown that the hemozoin knobs can be detected by magnetic resonance imaging.

**Conclusions:**

In the paper for the first time the characterisation of hemozoin pigment extracted from liver fluke *O. felineus* has been conducted. The role of hemozoin in the modification of immune response by opisthorchiasis is assumed.

**Electronic supplementary material:**

The online version of this article (doi:10.1186/s13071-015-1061-5) contains supplementary material, which is available to authorized users.

## Background

The feature of metabolism of parasites is the potential target for its detection and therapy. Hemozoin, also known as “malaria pigment”, is a byproduct, formed from the toxic heme moiety released during the digested hemoglobin detoxification. Therefore hemozoin formation is an adaptation to hematophagy. This pigment first identified and described for *Plasmodium falciparum* has been found in other *Plasmodium* species and in the series of blood-living parasite organisms: in the intracellular protozoan *Haemoproteus columbae* [1], in the trematodes *Schistosoma mansoni* [2, 3] and *Echinostoma trivolvis* rediae [4]. Moreover, hemozoin was found in insect ectoparasite *Rhodnius prolixus* [5].

Hemozoin is a unique biocrystal, a distinguishing trait of some blood-feeding organisms [6], it demonstrates high diagnostic and therapeutic potential. Hemozoin has been widely discussed as a candidate of target macromolecule for parasitic disease treatment [7–9]. The immunomodulate effects of hemozoin and especially its role in regulation of parasite-host interaction are also of a great interest [10, 11]. In general hemozoin is recognized as a key factor in the induction of malaria-associated immunosuppression [12, 13].

Nowadays, various approaches has been developed to diagnose parasitic infections (mainly malaria) based on hemozoin detection: light microscopy, polarization microscopy, RAMAN spectroscopy [14], chemo-luminescence method [15], flow cytometry [16] and mass spectrometry (MS) analysis [17, 18]. The unique physical properties of hemozoin have also been used as a basis for malaria diagnostic, e.g. photoacoustic detection [19, 20]. Thus magnetic properties of hemozoin have been widely used for magnetic separation of the malaria-infected cells [21–23]. Several diagnostic methods, such as magneto-optic technology test (MOT-test) [24–26], laser induced photothermal heating of hemozoin [6, 27], nuclear magnetic resonance and magnetic resonance relaxometry [28, 29] have been developed on the basis of magnetic properties of hemozoin crystal.

*Opisthorchis felineus* is a parasitic trematode widespread in Europe and Russia [30], especially in Western Siberia. This liver fluke settles in humans’ bile duct and causes the opisthorchiasis similar to the *O. viverrini* in South-East Asia. *O. felineus* infects a large number of people with potential fatal consequences. In some areas of Western Siberia (e.g. Tomsk region) up to 32.8 % of the population is infected [31]. Nevertheless, metabolites of *O. felineus* have been poorly studied.

During our study of liver fluke (*O. felineus*) infection in hamster model we have observed bile duct ecstasias filled by black-brown pigment. In this paper we provide the evidence that the identified pigment is a hemozoin identical to byproduct of *O. felineus*. Thus, the accumulation of the hemozoin in bile duct of *O. felineus* infected liver leads to the formation of knobs which can be detected by magnetic resonance imaging (MRI).

## Methods

Calcium chloride, sodium hydrogen carbonate, sodium chloride, hematin, sodium hydroxide, sodium dodecyl sulfate (SDS), phosphate buffer saline (PBS, tablet), Triton X-100, dimethyl sulfoxide (DMSO) were purchased from Sigma-Aldrich. Tris (Amresco), EDTA (AppliChem) were applied. All chemicals were used as received. Milli-Q (Millipore) water was used for all experiments.

### Experimental opisthorchiasis model

Metacercariae of *O. felineus* were obtained from naturally infected fish caught from fresh water reservoirs in endemic areas of Western Siberia (Tomsk), Russia. The muscular tissue and the subcutaneous tissue were digested by pepsin-HCl. Viable metacercariae were collected and identified by microscopy. Hamsters *Mesocricetus auratus* were purchased from the Department of Breeding and maintenance of small laboratory rodents of the Institute of Bioorganic Chemistry Academicians M.M. Shemyakin and U.A. Ovchinnikov. For conducting the experiment 6- to 8-week-old male hamsters were infected intragastrically with 50 metacercariae per hamster. Hamsters were housed five in each cage under conventional conditions and were fed with a stock diet and water ad libitum. Ethical Approval: All experiments and the maintenance of experimental animals were performed according to the guidelines of local Ethics Committee of the Siberian State Medical University (No. 3808 from 15.09.2014).

The infected animals (*n* = 4) were sacrificed at 5, 24 and 48 weeks postinfection, uninfected animals (*n* = 4) were used as control on each stage. Animals were euthanized by deep anesthesia with carbon dioxide. Four lobes of the liver were examined for the presence of ecstasia with black-brown pigment, followed by the histological analysis.

To characterize the pigment (hemozoin) of the adult worms, we collected samples of liver tissues avoiding liver fluke habitat areas and samples of bile ducts with pigment from six uninfected (control) and six infected hamsters at the 5 week postinfection.

### Histological analysis

The liver tissue or fluke was placed into 10 % buffered formalin, and then embedded in paraffin. Tissue sections were cut into 4–5 μm-thick slices and stained with hematoxylin and eosin. Histological analysis was performed with the optical microscope Axiostar plus (Carl Zeiss, Germany).

### Hemozoin extraction

To extract hemozoin from tissues approximately 30 mg of tissue was homogenized with the glass beads Precellys Ceramic Kit (Bertin technologies, Belgium) in five volumes of a solution containing 50 mM tris/HCl pH 8.0, 5 mM CaCl_2_, 50 mM NaCl and 1 % Triton X-100. To extract hemozoin from a fluke, the same procedure was used for one fluke. The homogenate was supplemented with 1 % Proteinase K, incubated overnight at 37 °C and centrifuged at 11,000 g for 40 min. The supernatant was discarded, the pellet was washed in 2 % SDS, 10 mM tris, pH 7.5 and then in 100 mM NaHCO_3_, pH 9.0 and roughly washed in the water with subsequent centrifugation for 10,000 g for 15 min [15].

### Determination of hemozoin concentration

To determine hemozoin concentration the sample was diluted in 100 mM NaOH, 2 % SDS, 3 mM EDTA and analyzed by spectrophotometric method. Series of hematin (10 μM – 1.2 nM) in 100 mM NaOH, 2 % SDS and 3 mM EDTA was used as a standard. The unknown hemozoin concentration was calculated from the calibration curve of the hematin concentration (nM) vs optical density (λ = 401 nm) [15]. The amount of hemozoin was expressed as nmol (hematin)/mg tissue or nmol (hematin)/fluke.

### *O. felineus in vitro* incubation

Adult (5 weeks postinfection) *O. felineus* flukes were taken from the bile ducts of hamster and incubated for 2 h in RPMI 1640 culture medium (PanEco, Russia) at 37 °C in an atmosphere of 5 % CO_2_. Adult worms were placed in single wells of 12-well plates (SPL Life Science) containing 3 ml RPMI 1640, supplemented with 1 % (vol/vol) antibiotics (50 mkg/ml streptomycin and 50 U/ml penicillin; PanEco, Russia) and incubated for 72 h [32].

### Fourier transform infrared spectroscopy (FTIR)

IR spectra were recorded on a FTIR-spectrometer Spectrum One B with diffusion refraction accessory (DRA) (Perkin Elmer, USA) in the range of 4000–400 cm^−1^ with 128 scans and at 4 cm^−1^ resolution.

### MS analysis

The extracted hemozoin or commercial hematin (C_34_H_32_N_4_O_5_Fe) samples were dissolved in 0.04 mL of DMSO, diluted in 0.9 mL of methanol. The samples were injected using a syringe pump (model 100, KD Scientific Inc., Holliston, MA, USA) at a constant flow rate of 0.24 ml/h. Positive ion mass spectra from 500 to 3000 (m/z) were obtained on a maXis impact HD ultra-high resolution quadrupole time-of-flight mass spectrometer (Bruker Daltonik GmbH, Germany) equipped with a standard electrospray ionization (ESI) ion source. The Tuning Mix ES-TOF G 1969–85000 (Agilent Technologies) was used for mass calibration. The spectra were processed by Compass for otofSeries 1.7 (oTof Control 3.4; Bruker Compass Data Analysis 4.2).

### Transmission electron microscopy (TEM)

For TEM analysis the samples of *O.felineus* extracted hemozoin crystals were placed on carbon-coated Cu grid. The TEM and HRTEM (high resolution TEM) images of hemozoin crystals were obtained using transmission electron microscope JEM-2200FS (JEOL, Japan) with a Cs-corrector at 200 kV.

Biological samples (liver, bile duct or *O. felineus* fluke) were fixed in 4 % p-formaldehyde in Hanks buffer, pH 7.4. After fixation samples were post-fixed with osmium tetroxide, dehydrated in alcohols of increasing concentration and propylene oxide, and then embedded in the Epon-Araldite mixture. Ultrathin unstained sections were observed in a Jem-1400 (JEOL, Japan) TEM.

### МRI *in vitro* and *ex vivo*

All ^1^Н-MRI experiments were performed on horizontal tomographic scanner with a magnetic field intensity of 11.7 T (Bruker, Biospec 117/16 USR, Germany) equipped with a transmitter volume (500.3 MHz, with diameter of 72/89 mm, Bruker, Biospec, Germany) and a receiver surface (500.3 MHz, with the size of 123 × 64 × 31 mm, Bruker, Biospec, Germany) ^1^H coils. T1 and T2 relaxation maps of water hemozoin suspension phantoms were recorded in order to investigate the MRI-contrast properties of hemozoin. For *ex vivo* MRI the liver was extracted from hamster after gas euthanasia and placed in 50 cm^3^ polypropylene tube in PBS buffer. T1-weighted high resolution images of hamster liver (slice thickness 1 mm; field of view 4.0 × 4.0 cm; matrix 512 × 512 dots) were recorded by RARE (rapid with relaxation enhancement) with the pulse sequence parameters TR = 1.5 s, TE = 9.2 ms. The signal intensities of each ROI (region-of-interest) in the T1 map were measured, T1 relaxation time data were calculated automatically by Paravision 5.0. [33]. Ethical Approval: The experimental protocol has been approved by the Bioethics Review Committee of the Institute of Cytology and Genetics (No. 24 from 28.10.2014).

### Statistical analysis

Statistical analyses were performed using IBM SPSS Statistics for Windows, Version 21.0 (Armonk, NY: IBM Corp.). All parameters were treated as non-parametric data. The data was expressed as median and interquartile ranges. Independent data groups were compared through Mann–Whitney test. The P value below 0.05 was considered as significant.

## Results

### Histological analysis of the infected liver

**Fig. 1:**
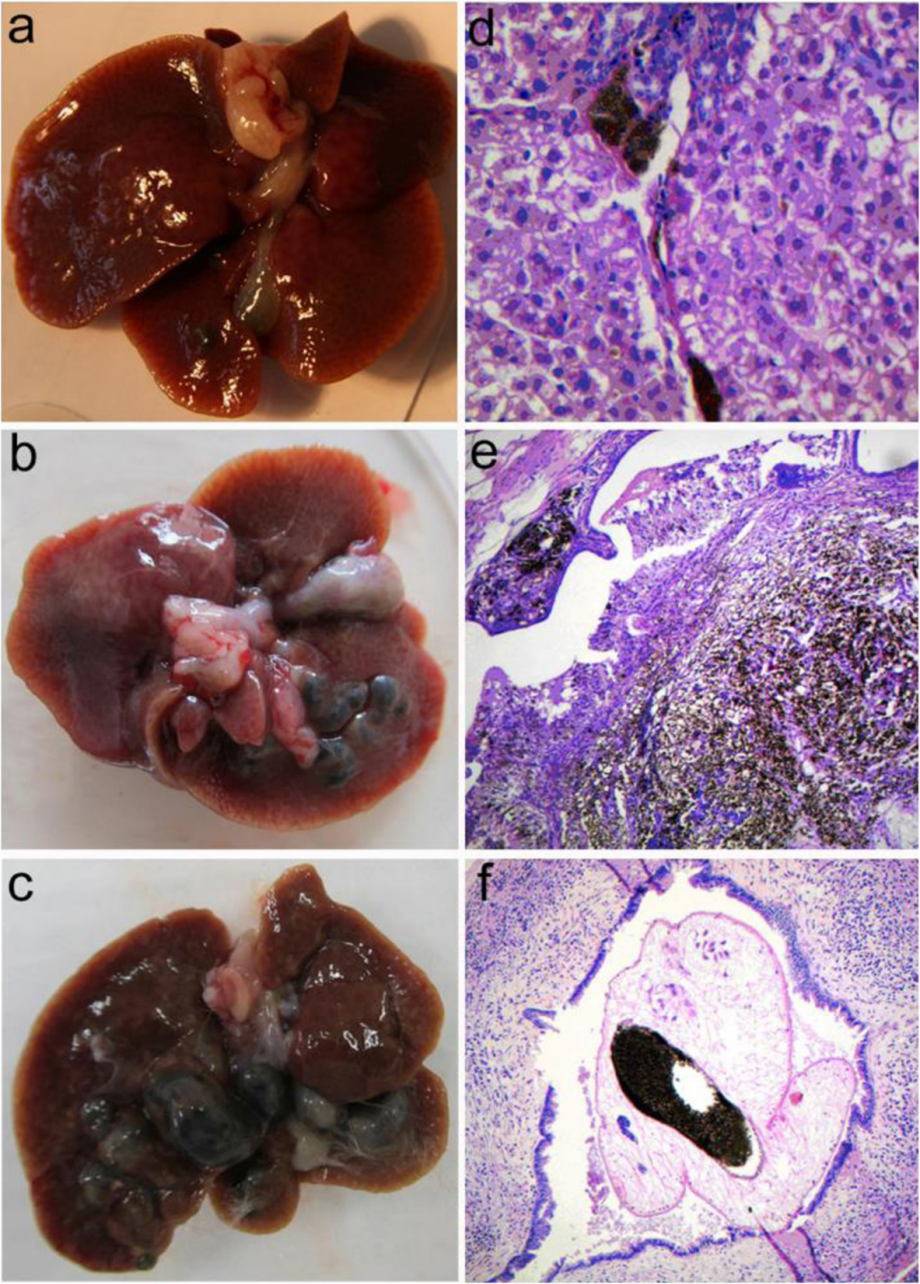
Histological analysis of the liver of *Opisthorchis felineus* infected hamster. Macrophotograph of the liver of *Opisthorchis felineus* infected hamster at (**a**) 5, (**b**) 24 and (**c**) 48 weeks postinfection. The black-brown pigment (hemozoin) forms the increasing hemozoin knobs. Histological analysis evidenced the hemozoin congestions in (**d**) small bile duct, hematoxylin and eosin staining (×400), (**e**) gallbladder wall and polypoid lesions of the gallbladder, hematoxylin and eosin staining (×200) and (**f**) *O. felineus* gut, hematoxylin and eosin staining (×400)

The visual analysis of *O. felineus* infected liver showed that dark knobs - the bile duct ectasia filled by black-brown pigment - can be found starting from 5 week postinfection. These knobs increased in size in the liver of infected animals in time of infection (Fig. 1a-c ). It’s important to note that the size and number of the knobs varied within the samples of the experimental group. The morphological analysis showed that similar knobs can be visualised not only on the surface but also inside a liver. In the control group of uninfected hamster the formation of the knobs has not been observed. The histological analysis evidenced no pathological changes in the liver and bile duct of the uninfected hamster (Additional file 1: Figure. S1).

Histological analysis data demonstrates periductal fibrosis with inflammatory cell infiltration occurring around the bile ducts of the *O. felineus* infected liver. At the 48 week of infection the mighty pigment clumps in bile duct and gallbladder wall were observed in the liver of infected hamster (Fig. 1d, e). The great congestion of pigment in gallbladder wall leads to chronic cholecystitis with sharply expressed proliferation of the epithelial cells and to the formation of the gallbladder polyp. It is important to note that polyp stroma has been filled with the pigment (Fig. 1e). The same granular black-brown pigment was observed in guts of the fluke (Fig. 1f).

### Hemozoin identification

The bile duct ectasia samples, samples of non-damaged liver tissue and *O. felineus* flukes were extracted from experimental hamsters and then pigment was extracted according to the protocols for hemozoin purification. The pigment remained insoluble during SDS and sodium bicarbonate at washing steps and it had characteristic hemozoin UV-visible spectra (Additional file 1: Figure. S2). In 0.1 M NaOH the pigment demonstrated an absorption spectrum identical to monomeric heme [3].

**Fig. 2:**
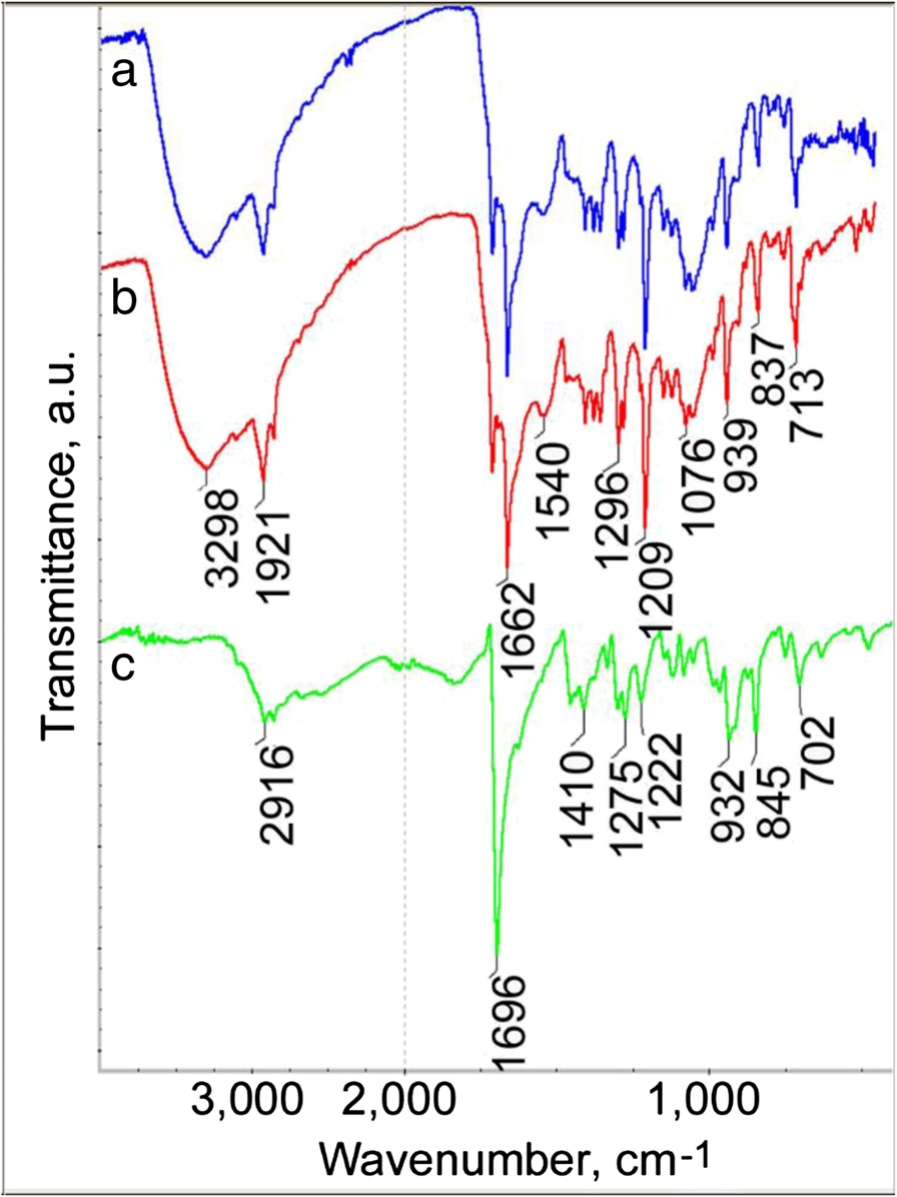
Fourier transform infrared spectroscopy (FTIR) of hemozoin. FTIR spectra of (**a**) hemozoin extracted from bile duct ectasia of infected hamster liver, (**b**) hemozoin extracted from *Opisthorchis felineus* and (**c**) commercial hematin (Sigma-Aldrich)

The pigment was also identified by MS and FTIR analysis as hemozoin. For the FTIR analysis the black-brown pigment was extracted from bile duct ectasia. On the FTIR spectra the characteristic hemozoin bands at 1662 and 1209 cm^−1^ related to С = O and C-O stretch vibration of the carboxylate group coordinated to the Fe (III) center were detected [34]. The FTIR spectra of hemozoin extracted from bile duct ectasia and *O. felineus* were identical (Fig. 2).

The Laser desorption mass spectrometry (LDMS) method is successfully used for hemozoin detection and identification in biological samples [4, 18, 35]. In these studies the low resolution mass-spectra of hemozoin were obtained via direct laser ultraviolet desorption. The mass-spectra exhibited a radical molecular cation (M+ with nominal mass 616 Da), corresponding to an individual ionized heme molecule, and several characteristic heme fragment ions.

**Fig. 3:**
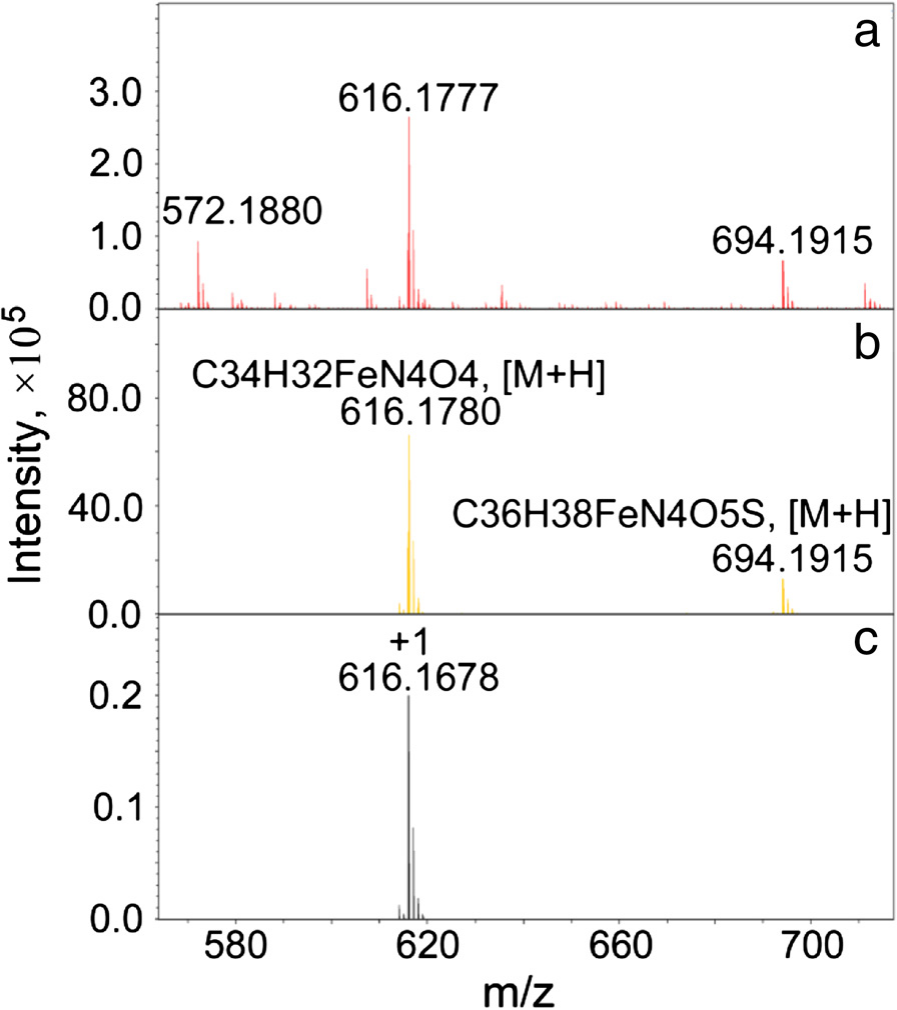
High resolution electrospray ionization (ESI) mass spectrometry analysis. The fragments of high resolution positive ion ESI mass spectra of (**a**) extracted from *Opisthorchis felineus* hemozoin in DMSO solution, (**b**) commercial hematin (Sigma-Aldrich) sample and (**c**) simulated mass-spectrum of [C_34_H_32_FeN_4_O_4_]^+^ ion

In our work the positive high resolution MS spectra were obtained using electrospray ionization (ESI) a so-called ‘soft ionization’ technique. Therefore the extensive fragmentation has not occurred. During the sample preparation the hemozoin was dissolved in DMSO to heme. In the mass-spectra of the sample the intensive molecular cation M+ at m/z 616.1777 with satisfactory accuracy corresponds to the expected molecular ion [C_34_H_32_FeN_4_O_4_]^+^ with calculated mass 616.1768 Da (Fig. 3). The mass-specter of hematin commercial sample (+616.1780) was similar. Also cluster peak with [C_34_H_32_FeN_4_O_4_ + DMSO]^+^ ions mass of +694.191 Da has been detected in both mass-spectra.

### Transmission electron microscopy (TEM) characterization of hemozoin

**Fig. 4:**
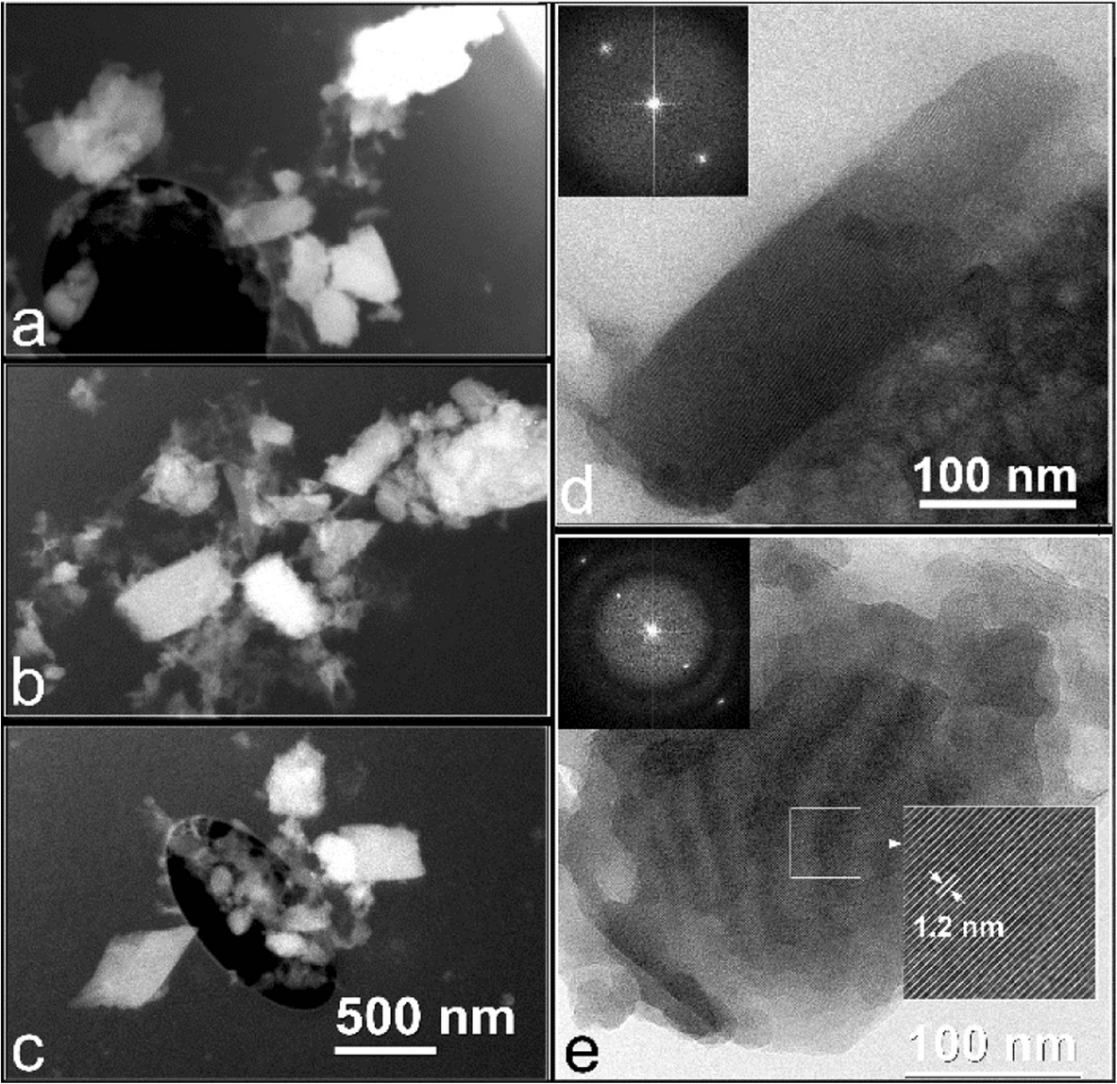
Transmission electron microscopy (TEM) analysis of extracted hemozoin crystals**.** (**a, b, c**) Dark field TEM images of hemozoin crystals extracted from *Opisthorchis felineus* (**d, e**) HRTEM image of hemozoin crystals, corresponding FFT image (in the left insert box) and an enlargement of the region (in the right insert box), exhibited regular lattice fringes with a spacing of 12.0 Å, confirming that the crystals tended to lie on their {100} or {1̅00} faces

Hemozoin crystals produced by the *O. felineus* were lath shaped with lengths about 100–600 nm and average length-to-width ratio about 2.0. The corresponding Fast Fourier transform (FFT) image confirmed that the isolated hemozoin crystals exhibited very regular lattice fringes with a spacing of 12.0 Å emphasizing that the crystals tended to lie on their {100} or {1̅00} faces (Fig. 4 and Additional file 1: Figure. S3). The determined lattice spacing of hemozoin crystals produced by *O. felineus* is consistent with the corresponding for synthetic crystals and hemozoin crystals from *P. falciparum* (varied from 11.9 to 12.6 Å) [36]. Parameters of external size of extracted hemozoin crystals produced by *O. felineus* and produced by *P. falciparum* hemozoin are slightly different. The hemozoin crystals produced by *P. falciparum* have average length 500 nm (from 180 to 1400 nm) and length-to-width ratio 3.4. By no surprise since hemozoin crystals extracted from various species such as *P. falciparum*, *S. mansoni* and *H. columbae* also have different morphology [1].

### Hemozoin dynamics in *O. felineus*

**Fig. 5:**
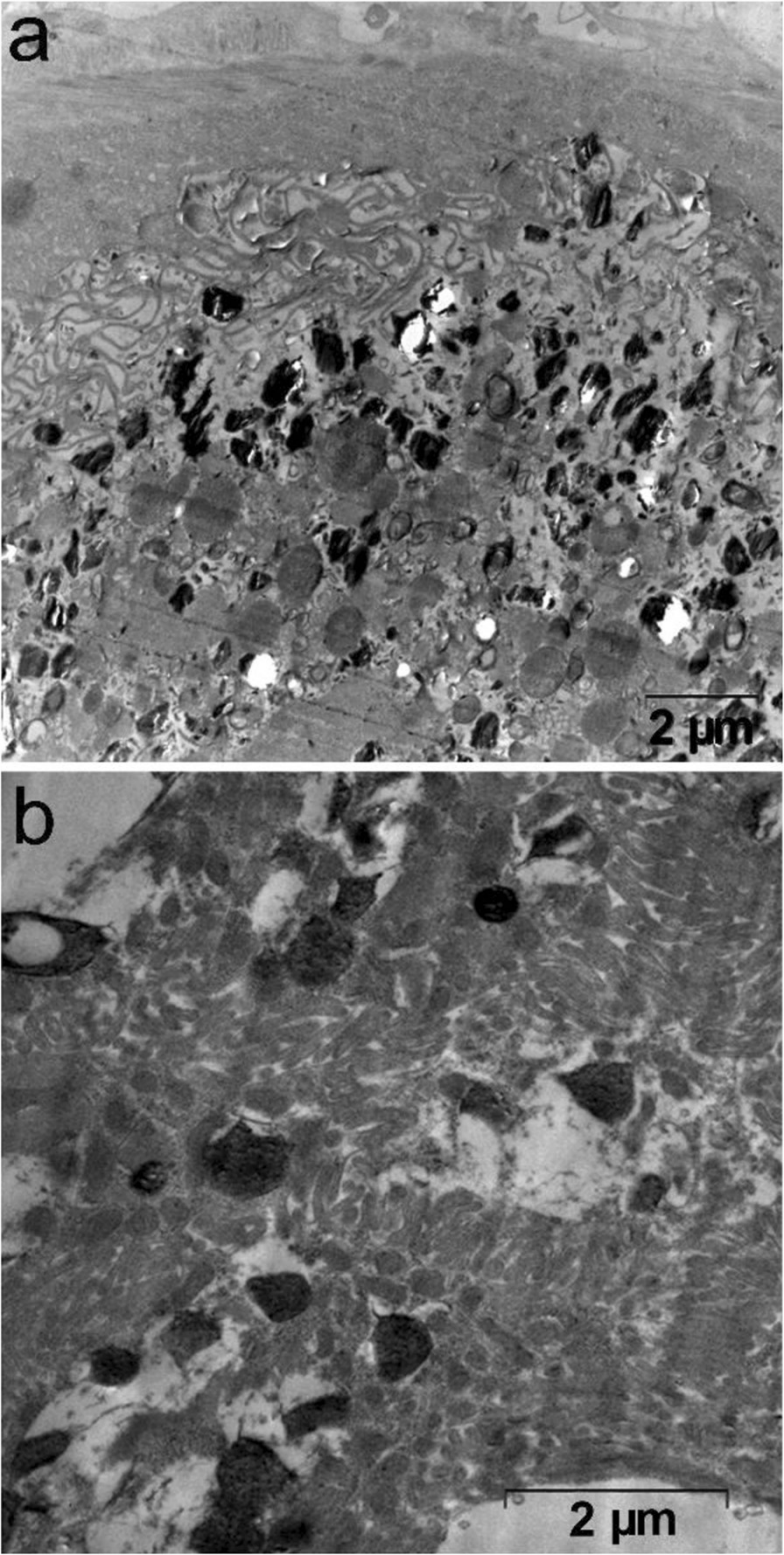
Transmission electron microscopy (TEM) analysis of hemozoin crystals observed *in vivo*. TEM images of hemozoin crystals in (**a**) *Opisthorchis felineus* gut and (**b**) bile duct lumen of *O. felineus* infected hamster (at 5 week postinfection)

In TEM examination of *O. felines* ultrastructure a large number of hemozoin crystals were found at the bottom of parasite gut and less in the oral sucker area (Fig. 5a). The average length of the hemozoin crystals was around 403 (232÷615) nm, however it varied from about 99–1024 nm (Additional file 1: Figure. S4), an average length-to-width ratio was equal to 2.0 (1.6÷2.6). No crystals were found in utherus, testes, vitelline, nor in a tegument of the fluke. It is important to note that hemozoin crystals were clearly observed in bile duct lumen of the infected animals (Fig. 5b).

The ability of the fluke to regurgitate hemozoin crystals in hosts’ bile duct leads to its accumulation in the parasite habitat area and formation of hemozoin knobs. The spectrophotometry revealed that the concentration of hemozoin in the knobs was 73-fold higher than in the liver parenchyma - 821.1 (536.4÷934.0) and 8.4 (7.7÷13.2) nmol (hematin)/g, respectively (*p* = 0.028).

**Fig. 6:**
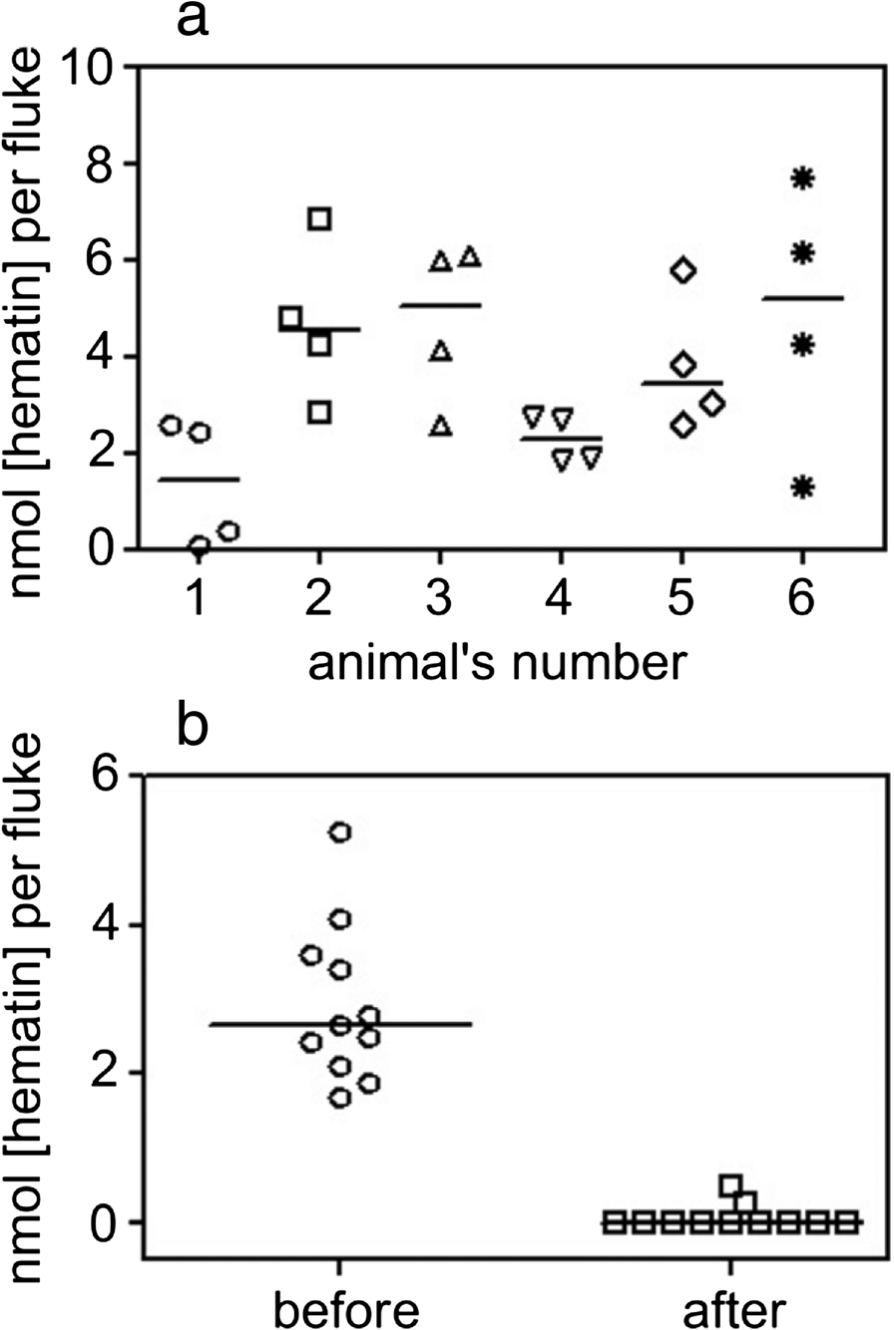
The hemozoin concentration in *Opisthorchis felineus* fluke. The hemozoin concentration in *Opisthorchis felineus* extracted from (**a**) six infected hamster at 5 week postinfection and (**b**) an infected hamster liver at 5 week postinfection before and after 3-days *in vitro* incubation in RPMI (*p* < 0.0001). The Mann–Whitney test was applied

Obviously, the concentration of hemozoin in parasite is not constant. Distribution of the hemozoin in adult fluke extracted from the liver of 6 infected hamsters is shown in Fig. 6a. The calculated median of hemozoin concentration in *O. felineus* was equal to 3.9 (2.07÷5.09) nmol (hematin) per fluke according to spectrophotometry. It was noted that this parameter varied from 0.08 to 7.66 nmol per fluke.

The ability of hemozoin to be released was clearly evident in the cultural experiments. The flukes removed from an infected liver were divided into two groups (*n* = 11); one of them was incubated in RPMI medium for 3 days. After incubation *in vitro* the hemozoin was practically absent in the preincubated flukes (*p* < 0.0001) (Fig. 6b). Thus the fluke loses the hemozoin during the incubation. This fact is important to consider during any investigation of a parasite or during their extract. It is recommended to exclude the worm preincubation step bearing in mind that hemozoin possess unique properties and contribute to the immune response.

### *Ex vivo* liver MRI

**Fig. 7:**
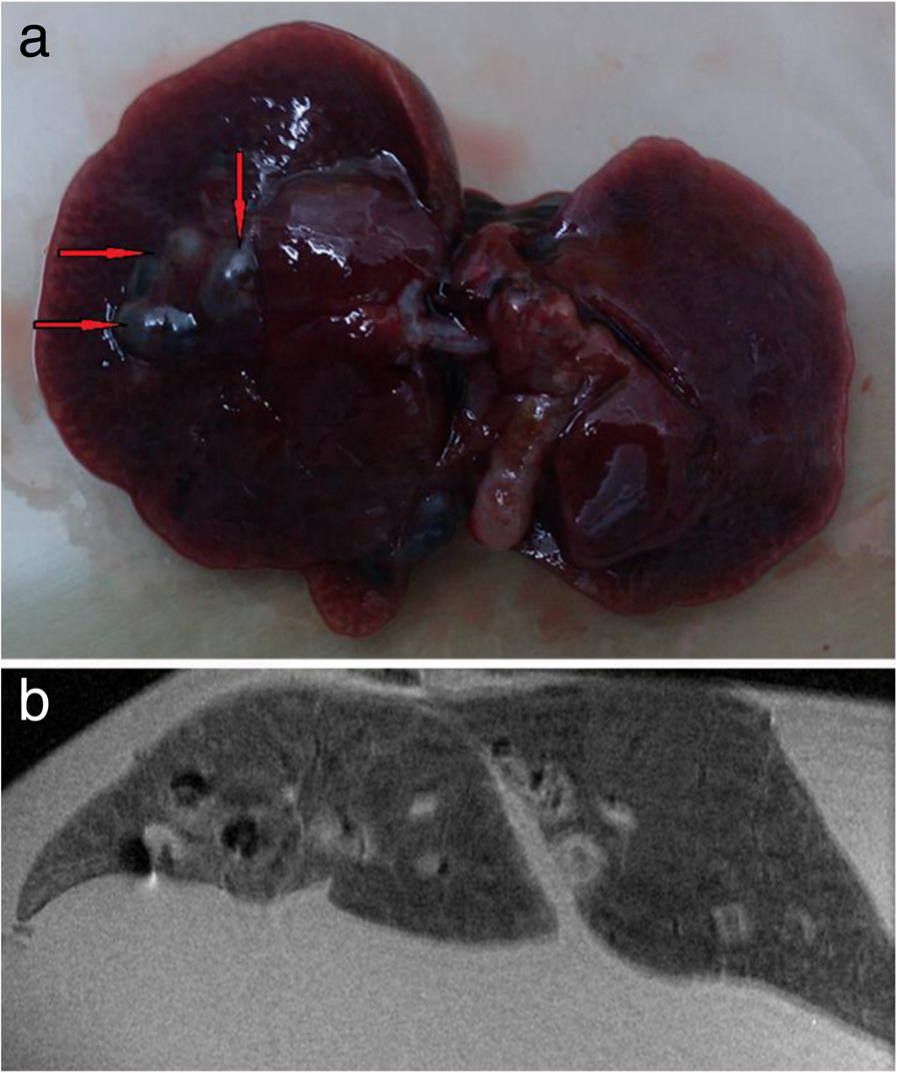
Detection of hemozoin knobs by magnetic resonance imaging. (**a**) The macrophotograph of *Opisthorchis felineus* infected hamster liver at 12 week postinfection. The hemozoin knobs in bile duct are marked by pointer; (**b**) *ex vivo* MRI of this liver in PBS buffer. The hemozoin knobs are visualised as dark areas

In MRI phantom experiments of water suspension of the extracted hemozoin we have not observed any contrast properties in both T1-and T2-weighted sequences. Although the *ex vivo* MRI of the *O. felineus* infected liver clearly detected significantly decreasing intensity of the signal on T1-weighted image in hemozoin concentration zone (Fig. 7). In hemozoin knobs T1 values are 5-fold shorter than T1 of liver parenchyma. These are equal to 279 msec and 1401 msec, respectively. Thus, hemozoin knobs can be detected as dark areas in the liver tissue in T1-weighted MRI scans.

We suppose that weaker MRI signal in the zone of hemozoin accumulation in comparison with surrounding tissue is detected due to high hydrophobicity of the pigment. This feature can be used for MRI visualisation of hemozoin clumps for diagnostic of the parasitic diseases.

## Discussion

*O. felineus* fluke produces hemozoin similar to other blood-feeding parasites. Previously Sripa and co-workers have observed black-brown pigment in the gut of the adult worm *O. felineus*. They have assumed that it can be an acid hematin, bilirubin, or even melanin [37].

It is important to emphasize that *O. felineus* rapidly regurgitated hemozoin crystals. It is well known that blood-living parasite ejects hemozoin in a host bloodstream. In bloodstream hemozoin is captured by macrophages migrating through the body and is accumulated in different organs [15]. The hemozoin is often observed in a liver and a spleen, less in lungs and kidneys during malaria infection [38]. The ability of parasites to regurgitate the hemozoin in blood circulation of host with subsequent accumulation in a liver is described for *S. mansoni* [11]. In the case of opisthorchiasis the hemozoin is accumulated in hosts’ bile duct and leads to hemozoin knobs formation. These hemozoin clumps cause the bile duct occlusion, mechanical damage and inflammation (cholecistitis), stimuli proliferation and neoplastic deformation (polyp). The fact of hemozoin detection in gallbladder provides a new insight in the pathogenesis of the gallbladder disease associated with *O. felineus* infection.

Olivera and co-workers earlier emphasized that hemozoin may play an essential role in the host-protective granulomatous response modulating and moderating [11]. On the other hand, *O. felineus* is able to modify the host immune response, namely, the opisthorchiasis infection diminishes genetic risk of atopic bronchial asthma [39] and prevents the food sensabilization [40]. Additionally, the monocyte-derived dendritic cells from peripheral blood of bronchial asthma patients stimulated with *O. felineus* extract *in vitro* exhibit decreased expression level of the most important costimulatory molecules CD86 on the cell surface [41]. Taking into account the immunosuppressive properties of hemozoin [13] and its ability to inhibit human monocytes differentiation and maturation [42], we supposed that the hemozoin production and its excretion can be the drive factor of the immunoregulatory activity of *O. felineus*. Considering the above, the key question for further research is a way by which the hemozoin crystals could pass a bile duct and be captured by macrophages.

## Conclusions

In summary, we have demonstrated that the liver fluke *O. felineus* produced a pigment hemozoin specific for some blood-feeding parasites and regurgitated it in a bile duct of host organism. This process leads to formation of massive hemozoin clumps so-called hemozoin knobs. The hemozoin knobs can be detected by T1-weigthened MRI. Taken into account the immunosuppressive properties of hemozoin, we can assume that this biomolecule plays a key role in modification of an immune system response during *O. felineus* infection. Thus, the investigation of hemozoin effects on immune system of *O. felineus* infected persons especially in the view of influencing on the progress of comorbidity immunopathology disease is very important.

## Additional file

Additional file 1:
**Hemozoin “knobs” in** ***Opisthorchis felineus*** **infected liver.** **Figure S1.** Histological analysis of the liver of uninfected hamster (control). Macrophotograph of the liver of uninfected hamster (control) at (**a**) 5, (**b**) 24 and (**c**) 48 weeks after the start of the experiment. Histological analysis of the liver of uninfected hamster (control) at (**d**) 5, (**e**) 24 and (**f**) 48 weeks after the start of the experiment, hematoxylin and eosin staining (×200). **Figure S2.** UV-visible spectra of hemozoin (in water) extracted from *Opisthorchis felineus* (1) and the same amount of hemozoin dissolved in NaOH, SDS, EDTA buffer. **Figure S3.** The intensity profile of the designated area in Fig. 2e in article. **Figure S4.** The histogram of hemozoin crystals length in *Opisthorchis felineus* gut (PDF 529 kb)

## Abbreviations

SDS: (Sodium dodecyl sulfate)
PBS: (Phosphate buffer saline)
DMSO: (Dimethyl sulfoxide)
EDTA: (Ethylenediaminetetraacetic acid)
FTIR: (Fourier transform infrared spectroscopy)
MS: (Mass spectrometry)
LDMS: (Laser desorption mass spectrometry)
DRA: (Diffusion refraction accessory)
TEM: (Transmission electron microscopy)
HR: (High resolution)
FFT: (Fast Fourier transform)
MRI: (Magnetic resonance imaging)
