# Supplementary material for: Hemozoin “knobs” in Opisthorchis felineus infected liver
Source: Parasit Vectors. 2015 Sep 17;8:459. doi: 10.1186/s13071-015-1061-5 (PMC4574221; doi:10.1186/s13071-015-1061-5)
Supplement: Additional file 1: — Hemozoin “knobs” in Opisthorchis felineus infected liver. Figure S1. Histological analysis of the liver of uninfected hamster (control). Macrophotograph of the liver of uninfected hamster (control) at (a) 5, (b) 24 and (c) 48 weeks after the start of the experiment. Histological analysis of the liver of uninfected hamster (control) at (d) 5, (e) 24 and (f) 48 weeks after the start of the experiment, hematoxylin and eosin staining (×200). Figure S2. UV-visible spectra of hemozoin (in water) extracted from Opisthorchis felineus (1) and the same amount of hemozoin dissolved in NaOH, SDS, EDTA buffer. Figure S3. The intensity profile of the designated area in Fig. 2e in article. Figure S4. The histogram of hemozoin crystals length in Opisthorchis felineus gut (PDF 529 kb) [file 13071_2015_1061_MOESM1_ESM.pdf]

Supplementary material

Hemozoin "knobs" in *Opisthorchis felineus* infected liver

Alexandra G. Pershina#, Irina V. Saltykova, Vladimir V. Ivanov, Ekaterina A. Perina, Alexander M. Demin, Oleg B. Shevelev, Irina I. Buzueva, Anton K. Gutakovskii, Sergey V. Vtorushin, Ilya N. Ganebnykh, Victor P. Krasnov, Alexey E. Sazonov, Ludmila M. Ogorodova

#Address correspondence to A.G. Pershina, allysyz@mail.ru

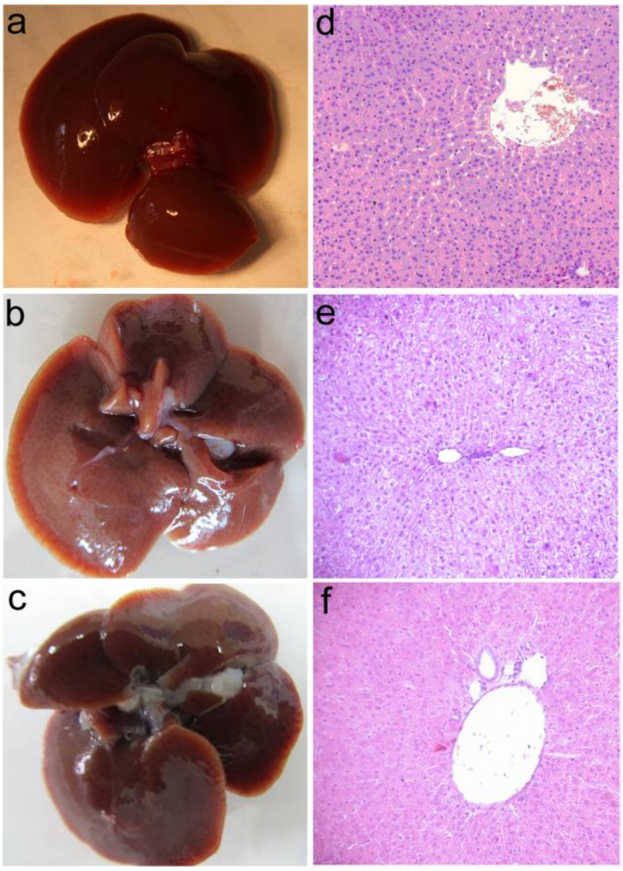

Supplementary Figure 1. Histological analysis of the liver of uninfected hamster (control). Macrophotograph of the liver of uninfected hamster (control) at (**a**) 5, (**b**) 24 and (**c**) 48 weeks after the start of the experiment. Histological analysis of the liver of uninfected hamster (control) at (**d**) 5, (**e**) 24 and (**f**) 48 weeks after the start of the experiment, hematoxylin and eosin staining (×200).

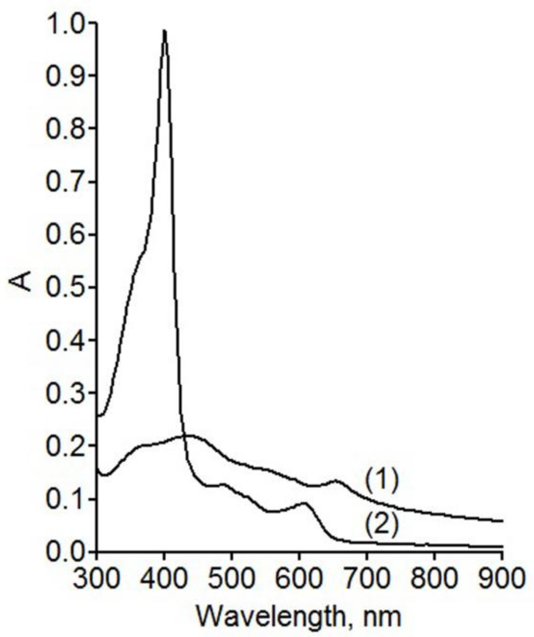

Supplementary Figure 2. UV-visible spectra of hemozoin (in water) extracted from *Opisthorchis felineus* (1) and the same amount of hemozoin dissolved in NaOH, SDS, EDTA buffer**.**

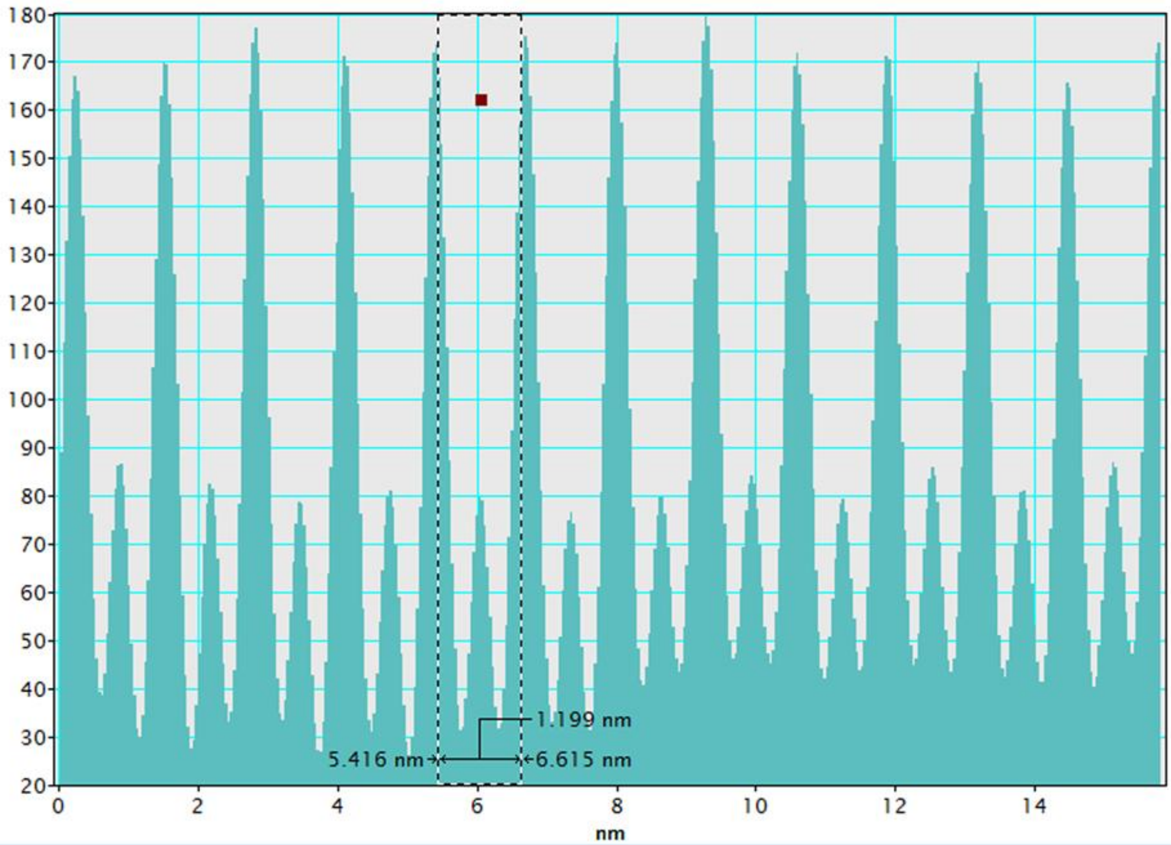

Supplementary Figure 3. The intensity profile of the designated area in Figure 2e in article.

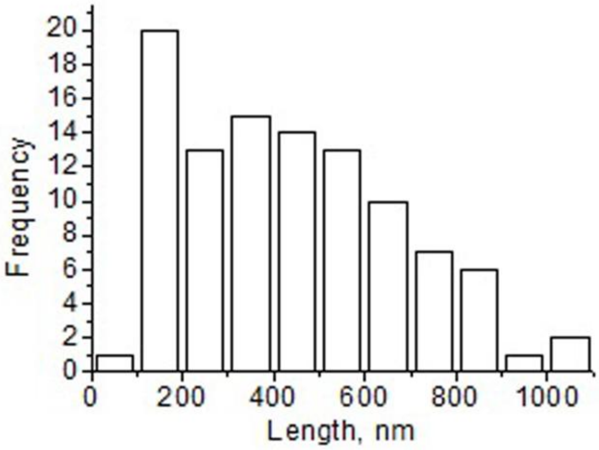

Supplementary Figure 4. The histogram of hemozoin crystals length in *Opisthorchis felineus* gut.
